# Current challenges in xenotransplantation

**DOI:** 10.1097/MOT.0000000000001146

**Published:** 2024-04-16

**Authors:** Marta Vadori, Emanuele Cozzi

**Affiliations:** a Department of Cardiac, Thoracic, Vascular Sciences and Public Health, University of Padua; b Transplant Immunology Unit, Department of Cardiac, Thoracic, Vascular Sciences and Public Health Padua University Hospital, Padua, Italy

**Keywords:** antibody mediated rejection, biosafety, genetically engineered pigs, xenotransplantation

## Abstract

**Purpose of review:**

In recent years, the xenotransplantation science has advanced tremendously, with significant strides in both preclinical and clinical research. This review intends to describe the latest cutting-edge progress in knowledge and methodologies developed to overcome potential obstacles that may preclude the translation and successful application of clinical xenotransplantation.

**Recent findings:**

Preclinical studies have demonstrated that it is now possible to extend beyond two years survival of primate recipients of life saving xenografts. This has been accomplished thanks to the utilization of genetic engineering methodologies that have allowed the generation of specifically designed gene-edited pigs, a careful donor and recipient selection, and appropriate immunosuppressive strategies.

In this light, the compassionate use of genetically modified pig hearts has been authorized in two human recipients and xenotransplants have also been achieved in human decedents. Although encouraging the preliminary results suggest that several challenges have yet to be fully addressed for a successful clinical translation of xenotransplantation. These challenges include immunologic, physiologic and biosafety aspects.

**Summary:**

Recent progress has paved the way for the initial compassionate use of pig organs in humans and sets the scene for a wider application of clinical xenotransplantation.

## INTRODUCTION

The limited availability of human organs, tissues and cells remains a serious barrier to the broader application of transplantation medicine. Worldwide the gap between the demand for organs and the number of organs available is considerable and continuously increasing [1]. This has encouraged the scientific community to investigate alternative approaches including transplantation of organs, tissues and cells between different species, referred to as xenotransplantation.

Due to its anatomical and physiological similarities with man, the short gestation period and its rapid growth, possibly resulting in an unlimited supply of organs of any size, the pig is viewed as the most suitable species for the clinical application of xenotransplantation. In addition, genetic engineering provides the opportunity to modify the pig organs and improve their immunological and physiological compatibility with man.

This short review will focus on the research challenges and the recent results obtained in the field of kidney and heart xenotransplantation. The considerable advancements of preclinical research in the area of solid organ xenotransplantation have allowed the initiation of the first clinical applications in humans. In this article, particular attention will be paid to the immunology, physiology and safety aspects of pig-to-primate xenotransplantation. 

**Box 1:**
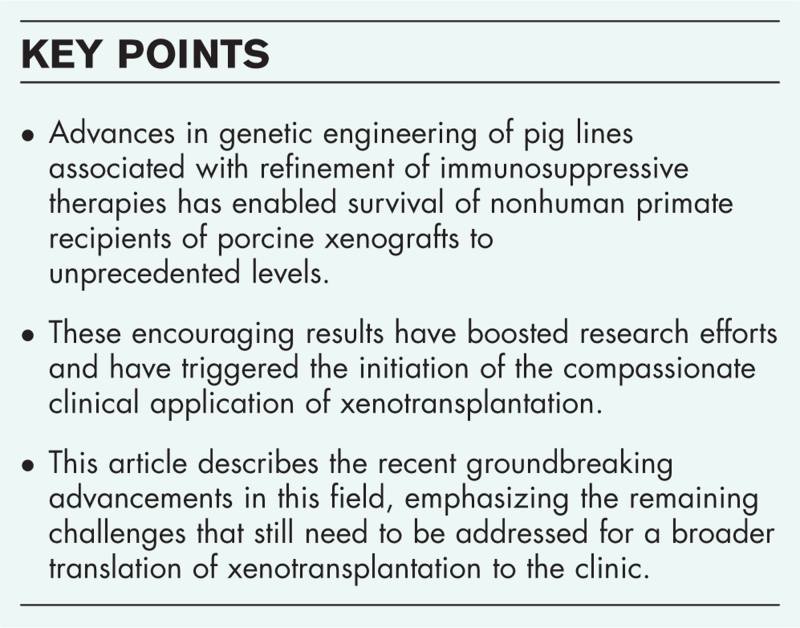
no caption available

## IMMUNOLOGICAL CHALLENGES

With regard to the immunological obstacles to the successful clinical application of xenotransplantation, the current situation can be summarized as follows.

### Antibody-mediated rejection

The humoral component of the immune response has always been considered as the main barrier to the long-term survival of xenotransplanted organs and, to date, it has never been consistently overcome in pig-to-primate models. In this context, 3 glycans, namely αGal antigen (Galα(1,3)Galβ4Glc-Nac-R), the Neu5GC antigen (*N*-glycolylneuraminic acid) and the SdA (Sia-α2.3-[GalNAc-β1.4]Gal-β1.4-GlcNAc) represent the principal targets of the humoral immune response [2,3].

The advent of CRISPR/Cas9 technology, a highly precise, effective and easy-to-use genome editing technique has enabled the generation of pigs knock out for each of the enzymes involved in the production of the above-mentioned sugar moieties, the so-called triple knock-out donor pigs (TKO). Different studies have shown that humans may have preformed antibodies against TKO pigs [4,5]. Such antibodies in part recognize the histocompatibility swine leukocyte antigens (SLA) possibly due to a cross-reaction with homologous epitopes on the HLA class I molecules [4]. Indeed, Martens and colleagues have clearly demonstrated that anti-HLA antibodies primarily directed against HLA locus-A molecules may recognize porcine SLA antigens [4]. In particular, it has been demonstrated that up to 27% of patients waiting for a renal transplant have anti-SLA I antibodies (either IgM or IgG) [6]. Similarly, anti-HLA class II antibodies may recognize SLA DR and DQ antigens [7]. Furthermore and unexpectedly, anti-SLA antibodies may be detected in a small percentage of HLA nonsensitized patients [6].

Although a recent survey regarding the outcome of kidney xenografts in nonhuman primate (NHP) has highlighted that antibody-mediated rejection represents the first cause of graft loss in the first year (45% of cases) [8], a tremendous improvement in long-term survival of xenograft recipients has been achieved.

Of note, survival exceeding 6 months has also been reported following transplantation of life supporting solid organ xenografts, with a survival of up to 758 days for a porcine kidney recipient [9^▪▪^] and 264 days for a pig heart transplanted into primate [10^▪^]. The prevention of early antibody-mediated rejection appears to be achievable with: (i) careful recipient selection i.e. recipients with low preexisting antidonor xenoantibodies [9^▪▪^,^11^,^12^,^13^]; (ii) a multiple gene editing of the porcine donor, which must include the deletion of the three major glyco-antigens and the insertion of [at least] one human complement-regulatory protein, such as CD46, CD55 or CD59; (iii) a refinement of the immunosuppressive protocol with an induction therapy with B- and T-cells depleting agents, and a maintenance treatment with co-stimulatory blocking agents.

Currently, there is no standard immunosuppressive regimen applied in preclinical pig-to-primate xenotransplantation studies. However, based on the best results achieved in recent NHP studies, a regimen based on an induction with antithymocyte globulin, rituximab, and C1 esterase inhibitor, and a maintenance therapy with an anti-CD154 monoclonal antibody (mAb), rapamycin, methylprednisolone and tocilizumab has been proposed as a possible regimen to prevent the onset of antibody-mediated rejection in preclinical studies [14^▪^].

### Cell-mediated rejection and inflammation

Solid organ xenotransplantation in NHP also triggers a cell-mediated immune response often due to the existence of receptor incompatibilities between species, leading to dysregulation of the activation/inhibition signals that finely modulate the activity of innate and adaptive immune cells.

In the case of innate immunity, it has been shown that human natural killer (NK) cells infiltrate rapidly pig xenotransplants and damage the graft via both direct and indirect mechanisms. Activation of NKp44 or NKG2D receptors on human NK cells by ligands expressed on pig cells associated with nonrecognition of porcine SLA-1 molecules by human NK inhibitory receptors, leads to direct cell-mediated lysis of pig cells by release of granzymes and perforin [15]. Uncertainty remains on the porcine molecules triggering direct cytotoxicity by human NK cells against genetically modified pig endothelial cells. Additionally, the role of the porcine ULBP-1 molecule, which was previously considered to be the primary trigger for NK cell activation, is now being challenged [16]. Indirect mechanisms of NK cell-mediated damage involve: (i) antibody mediated cell cytotoxicity (ADCC); (ii) induced-T cell activation via pro-inflammatory cytokine release [17]; (iii) production of xenoreactive antibodies via a T cell-independent mechanism by a CD40/CD154 costimulation of B cells in the marginal area of the spleen [18]. The use of genetic engineering to express on pig cells inhibitory ligands such as nonclassical human leukocyte antigens HLA-E and -G can be a strategy to overcome NK responses in xenotransplantation. Indeed, at least in vitro, co-expression of both these HLA molecules on pig cells is associated with a significant reduction of human NK cell degranulation [19].

A fundamental role in the rejection of a xenograft is played by monocytes and macrophages that contribute with their phagocytic activity. Also in this case, the lack of compatibility between the human macrophage inhibitory receptor SIRP-α and CD47 on pig cells promotes phagocytic activity of macrophages [20]. The use in preclinical studies of genetically engineered pigs that also express hCD47 is believed to contribute to the long-term xenograft survival achieved to date [10^▪^].

Possible approaches to mitigate protracted inflammatory responses triggered by innate immune cells in xenografts could be the use of organs from genetically modified pigs expressing anti-inflammatory genes like human HO-1 [9^▪▪^,^21^] and A20 [22]. In this context also the use of anti-inflammatory agents such as tocilizumab, a monoclonal antibody against the human receptor for the proinflammatory interleukin (IL)-6, or etanercept, a tumor necrosis factor (TNF)-α inhibitor, appears to be associated with beneficial effects and has been suggested in the early post-transplant period as a means to counteract the inflammatory response, the induced T cell response and antibody production [10^▪^,14^▪^].

T cells are likewise involved in the rejection of a xenotransplant. Human T cells can react against pig cells by directly recognizing SLA molecules or can indirectly recognize processed xenoantigens presented by self-MHC. The recognition of the antigen in an indirect xenogenic context by CD4^+^ T lymphocytes appears to be more vigorous than in the allogeneic context, possibly due to the increased number of xenoantigens presented by human antigen presenting cells (APCs) [23]. In addition, it has been reported that the main xenogenic peptides indirectly recognized by T cells on human APCs derive from SLA molecules [23]. It has also been shown that SLA alleles are able to induce a very high cellular immune response [24]. Similarly, subjects with particular HLA allelic configurations were found to be strong responders to pig cells [25]. These data suggest that an accurate selection of the donor and recipient may be important to protect the graft from the humoral response but also from the cellular immune injury. In addition, an induction treatment aimed at depleting T cells, preferably CD4^+^ T cells [12], and a maintenance immunosuppression based on the use of blockers of the CD40/CD154 co-stimulation pathway appear fundamental. Likewise, the use of rapamycin, included by some in the preclinical immunosuppressive regimen, has been shown to block CD4^+^ T helper cells and cytotoxic T cells and increase the number of Tregs [26]. In addition, rapamycin reduces pro-inflammatory cytokines.

## PHYSIOLOGICAL CHALLENGES

Extensive preclinical testing conducted in relevant NHP models suggests that xenografts are sufficiently similar to their human counterpart to support the recipient physiological and functional needs. However, several physiological differences have been reported.

These include alterations of the coagulation cascade primarily related to: (i) endothelial cell activation; (ii) increased tissue factor expression on recipient's platelets; and (iii) molecular incompatibilities between donor and recipient factors involved in the regulation of the coagulation cascade. In particular, porcine TFPI does not efficiently block Factor Xa, porcine thrombodulin does not activate the anticoagulant protein C, and porcine von Willebrand factor causes excessive platelet aggregation in primates. These dysregulations of the coagulation cascade may result in thrombotic microangiopathy and consumptive coagulopathy in the recipient, often associated with bleeding disorders and, in many cases, in the presence of an antibody-mediated response [27]. In this light, pigs have been engineered to express human thrombomodulin and endothelial cell protein receptor C (EPCR) to reduce coagulative incompatibilities and thrombotic microangiopathy often observed in rejected kidney and heart xenografts [28].

As far as kidney xenotransplantation, an in-depth analysis of the physiological aspects has recently been reported [29]. In this regard, compared to the human counterpart, pig kidneys have the same anatomical organization but a lower percentage of long-looped nephrons ultimately resulting in a reduced ability to concentrate urine [30]. Preclinical studies, however, show that pig kidneys are able to maintain almost all electrolytes in a physiologically normal range and maintain functionality for a long time. Hypercalcemia and hypophosphatemia have been reported following TKO kidney xenotransplantation in NHP [26]. The mechanisms underlying this dysregulation are still unclear and necessitate further investigations in preclinical and clinical studies [29,31]. The glomerular filtration rate (GFR) and the blood flow are comparable between pig and man, as well as the level of proteinuria between the two species [32]. Therefore, all these parameters can be evaluated as markers of posttransplant kidney function. Nonetheless, differences in the activity of hormones that regulate renal function have been reported. In particular: (i) human angiotensinogen does not appear to be an optimal substrate for renin produced by pig kidney, suggesting that the renin–angiotensin–aldosterone system (RAAS) may be less effective after pig kidney xenotransplantation in human as recently demonstrated in the short-term clinical study in brain-death human recipients [33,34]; (ii) the human antidiuretic hormone (ADH) is structurally different from the porcine counterpart with reduced agonistic activity on the porcine ADH receptor, ultimately decreasing water reabsorption, as confirmed by Judd and colleagues [34]; (iii) although similar to human, porcine erythropoietin does not appear to adequately support erythropoiesis in the primate and may contribute to the anemia observed after transplantation in NHPs. Finally, it has been confirmed that porcine and human kidneys have similar abilities in renal clearance for both exogenous and endogenous substrates [34].

It has been reported that porcine kidneys and hearts grow rapidly following xenotransplantation and the use of pigs knock-out for the growth hormone receptor gene or the use of miniature pigs as source donors may avoid such an undesirable growth [9^▪▪^,^29^]. In the case of heart, it has been observed that life supporting porcine hearts transplanted in NHP develop concentric hypertrophy and significant left ventricular outflow tract obstruction [35]. This extensive myocardial hypertrophy may be the consequence of the higher pressure in NHP. Indeed, NHP and humans have significantly higher systemic vascular resistance and mean blood pressure than pigs. Therefore, a tight control of blood pressure will be necessary in order to avoid early heart failure [36]. Furthermore, the use of rapamycin, antihypertensive therapies and weaning from the steroid therapy may contribute to reduce cardiac overgrowth [37].

Mechanically, the pig heart is very similar to the human counterpart. Parameters of systolic output, cardiac output, mean blood pressure, heart rate and myocardial blood flow are comparable. Between the two species, however, there is a morphological difference of the atrioventricular node, with a consequent difference in the action potential of cardiomyocytes, which could lead to an increased risk for arrhythmias. Still, the long-term survival of porcine organs in heterotopic and orthotopic preclinical models shows that myocardial pig functionality is not impaired following transplantation in primate.

## BIOSAFETY

Xenotransplantation is associated with the risk of infections caused by both common human pathogens [such as cytomegalovirus (CMV) or Epstein–Barr virus (EBV)] and potential infectious agents of swine origin. In this case, pathogens that could be problematic are: (i) those remaining latent at the intracellular level in asymptomatic subjects such as the pig cytomegalovirus (pCMV), the pig lymphotropic herpes virus (PHLV), the hepatitis E virus (HEV) and endogenous porcine retroviruses (PERV) (ii) possibly unidentified microorganisms, a condition that requires continuous posttransplant monitoring [38,39^▪^]. pCMV and PHLV are two herpes viruses with ability to infect human cells. Infection by pCMV may cause endothelial cell activation and systemic coagulopathy in the transplanted organ and lead to xenotransplant rejection, while PHLV can cause lymphomas. Of note, pCMV infections are unequivocally linked to reduced xenograft recipient survival in preclinical models [40–42] and, incidentally, a pCMV reactivation was evidenced in the cardiac xenograft recipient recently reported by the University of Maryland [43^▪^]. As far as PERV, to date there is no evidence of PERV infections either in pig-to-nonhuman primate preclinical models or in the recently reported clinical cases [44–47].

It is noteworthy that a thorough screening of source animals before xenotransplantation procedures may, at least in theory, result in enhanced microbiological safety as compared to conventional human organ donation. In addition, by applying CRISPR/Cas9 genomic editing technique, it is possible to breed pig strains in which PERV sequences have been inactivated [9^▪▪^,^48^–^50^]. In any case, to reduce the risk of infections, xenotransplantation requires a thorough microbiological surveillance of donor animals raised in biosecure pig facilities, the so-called ‘designated pathogen-free’ animals (DPF) [39^▪^]. This designation refers to pig colonies where a list of potential human and pig pathogens have been excluded. In addition, the safety measures for xenotransplantation must include strict and lifelong monitoring of xenograft recipients and their close contacts for both swine and human pathogens, the collection of samples to be stored in biobanks for future investigations and the development and validation of highly sensitive serological and quantitative PCR tests to assess the possible presence of porcine viruses in human cells [50]. In this context, a multitesting approach based on a combination of serum-ELISA and nested-PCR, Western blot, immunofluorescence and qPCR has recently been proposed to improve surveillance of pCMV [51].

## RECENT CLINICAL STUDIES

In the last few years, the considerable advancements in survival of nonhuman primate recipients of life supporting xenografts have allowed the initiation of xenotransplantation studies in man.

The recent compassionate transplantation of a 10-gene-edited pig heart into two patients not eligible for an allograft represents a very important step forward. So far, only data regarding the first of these two cases have been reported [43^▪^]. The recipient received an immunosuppressive regimen based on rituximab, thymoglobulin, C1-esterase complement inhibitor, humanized anti-CD40 mAb, mycophenolate mofetil and steroids. Two infusions of human intravenous immunoglobulins (IvIg) were administered due to hypogammaglobulinemia. Following xenotransplantation, the graft functioned well until postoperative day 47 when diastolic heart failure occurred, associated with increased serum troponin I and histopathological findings of severe endothelial injury. At this stage, three potential causes of xenograft endothelial cell damage have been identified: (i) antibody-mediated rejection; (ii) exogenous administration human IvIg containing xenoantibodies possibly causing immune activation; (iii) reactivation and replication of latent porcine cytomegalovirus or porcine roseolovirus (pCMV/PRV) within the xenograft. Although the postoperative period was not without problems an exceptional survival of 60 days has been reported. A comprehensive postmortem evaluation allowed the authors to suggest the following recommendations in view of possibly future clinical studies: (i) a stringent patient selection process is fundamental; (ii) great attention should be paid to the immunosuppressive therapy; in particular a close monitoring of the levels of co-stimulation blockade antibodies is fundamental; in this case apparently the clearance of anti-CD40mAb was higher than predicted; (iii) the administration of IvIg should preferably be avoided; (iv) advanced monitoring techniques to exclude the presence of pCMV or any other pathogen should be implemented [52^▪▪^]. Indeed, despite close monitoring and the use of a pig raised in a biosecure facility, inadvertent transmission of pCMV to the patient could not be avoided. At this stage, we are aware that a second patient underwent cardiac xenotransplantation and his survival reached 42 days with rejection being the cause of death. The scientific community is very eager to read a detailed report on such a case [53].

As an alternative approach, two North American groups have xenotransplanted genetically modified pig kidneys or hearts into brain-dead human recipients [54]. Although not perfect, this ‘decedent model’ allows the acquisition of very valuable insight on the immunological, physiological and biosafety issues related to clinical xenotransplantation, providing interesting data that nicely complement those generated in preclinical NHP studies. In two cases, a GTKO pig thymo-kidney was transplanted into recipients immunosuppressed with mycophenolate mofetil and methylprednisolone. The study lasted for 54 h. Throughout the study the xenotransplanted kidneys remained well perfused, the GFR increased and they continued to produce abundant amounts of urine with decreased creatinine levels. The recent and comprehensive kidney evaluation, combining transcriptomics and multiimmunophenotyping histological analyses, evidenced an indolent and subclinical form of antibody-mediated rejection characterized by innate immune cells (macrophages, monocytes, neutrophils, and natural killer cells) infiltration, endothelium activation characterized by microvascular inflammation, increased expression of genes associated with antibody-mediated rejection and antibody deposition [55^▪▪^]. Similar studies in decedent recipients have also been conducted by others [47,56]. In these cases, recipients were nephrectomised and bilaterally transplanted with kidneys from engineered pigs with 10 genetic modifications. Patients received a conventional immunosuppression and the first case was monitored for 74 h during which urine production was poor and creatinine clearance did not improve. Histologically, a moderate-grade thrombotic microangiopathy was observed from the beginning; it did not progress over time and was not associated with antibody deposits, complement or cell-mediated rejection. In a second case, the anti-C5 complement inhibitor eculizumab was added to the induction immunosuppressive therapy and patient monitoring lasted for 7 days. Importantly, improved creatinine clearance was observed and no signs of thrombotic microangiopathy were reported [57]. A further clinical study in a recipient of a porcine kidney surviving beyond one month is underway and the related data are expected to be released in the near future. In addition, a short-term study (3 day survival) has also been conducted with 10-gene edited pig hearts orthotopically transplanted into 2 deceased recipients. In both cases, there was an excellent cardiac function immediately after transplantation, although cardiac function declined postoperatively in one case, possibly due to size mismatch between donor and recipient. In any case, the explanted hearts did not evidence cellular or antibody-mediated rejection [58].

## CONCLUSION

This review highlights the impressive steps forward recently accomplished by the preclinical and clinical xenotransplantation science. At this stage, several areas of research necessitate further progress and development: (i) there is the need to identify the ideal genetic engineering profile of the donor pig; (ii) the most adequate immunosuppressive protocol has yet to be defined; (iii) the current clinical experience highlights the need for developing sensitive microbiological tests to prevent and timely detect latent zoonotic infections. In addition, considerable investments to cover the important costs for maintenance of bio-secure herds appear indispensable. Indeed, a broader application of clinical xenotransplantation may not be too distant.

## Acknowledgements

*None.*

### Financial support and sponsorship

*None.*

### Conflicts of interest

*There are no conflicts of interest.*

## REFERENCES AND RECOMMENDED READING

Papers of particular interest, published within the annual period of review, have been highlighted as:

▪ of special interest
▪▪ of outstanding interest
